# Fucosylated haptoglobin is a novel predictive marker of hepatocellular carcinoma after hepatitis C virus elimination in patients with advanced liver fibrosis

**DOI:** 10.1371/journal.pone.0279416

**Published:** 2022-12-21

**Authors:** Kumiko Shirai, Hayato Hikita, Ryotaro Sakamori, Akira Doi, Yuki Tahata, Sadatsugu Sakane, Yoshihiro Kamada, Kazuhiro Murai, Akira Nishio, Ryoko Yamada, Takahiro Kodama, Yasutoshi Nozaki, Naruyasu Kakita, Hisashi Ishida, Fumihiko Nakanishi, Naoki Morishita, Kazuho Imanaka, Mitsuru Sakakibara, Tomohide Tatsumi, Eiji Miyoshi, Tetsuo Takehara

**Affiliations:** 1 Department of Gastroenterology and Hepatology, Osaka University Graduate School of Medicine, Suita, Japan; 2 National Hospital Organization Osaka National Hospital, Osaka, Japan; 3 Division of Health Sciences, Department of Advanced Metabolic Hepatology, Osaka University Graduate School of Medicine, Suita, Japan; 4 Kansai Rosai Hospital, Amagasaki, Japan; 5 Kaizuka City Hospital, Kaizuka, Japan; 6 Ikeda City Hospital, Ikeda, Japan; 7 National Hospital Organization Osaka Minami Medical Center, Kawachinagano, Japan; 8 Minoh City Hospital, Minoh, Japan; 9 Itami City Hospital, Itami, Japan; 10 Yao Municipal Hospital, Yao, Japan; 11 Division of Health Sciences, Department of Molecular Biochemistry and Clinical Investigation, Osaka University Graduate School of Medicine, Suita, Japan; University of Navarra School of Medicine and Center for Applied Medical Research (CIMA), SPAIN

## Abstract

**Background:**

Patients with advanced fibrosis are at risk for developing hepatocellular carcinoma (HCC) even after hepatitis C virus (HCV) elimination. We previously reported that serum fucosylated haptoglobin (Fuc-Hp) levels increase as the disease progresses from chronic hepatitis to cirrhosis and then HCC. However, it remains unclear whether serum Fuc-Hp levels can stratify the risk of HCC occurrence after a sustained virological response (SVR) is achieved with direct-acting antivirals (DAAs) in patients with advanced liver fibrosis.

**Methods:**

Among 3,550 patients with chronic hepatitis C treated with DAAs at Osaka University Hospital and related hospitals, the stored sera of 140 patients who were diagnosed with F3 or F4 by liver biopsy before DAA treatment, achieved SVR, and had no history of HCC were available at both baseline and the end of treatment (EOT). We measured the Fuc-Hp levels in these samples.

**Results:**

The median serum levels of Fuc-Hp at EOT were significantly lower than those at baseline. During the 54.4-month follow-up period, 16 of 140 patients developed HCC. Multivariate Cox proportional hazards analysis revealed that high Fuc-Hp at EOT, high body mass index (BMI), and low albumin at EOT were independent risk factors for HCC occurrence. Patients with all three factors—high Fuc-Hp, high BMI, and low albumin—had a higher incidence of HCC than patients without these factors.

**Conclusions:**

High serum Fuc-Hp levels at EOT were an independent risk factor for HCC occurrence after SVR. Combined with BMI and albumin, Fuc-Hp can stratify the risk of HCC occurrence among those with advanced fibrosis.

## Introduction

The widespread availability of direct-acting antivirals (DAAs) has dramatically changed the landscape of hepatitis C virus (HCV) therapy. Treatment with DAA is well tolerated, and patients with cirrhosis as well as chronic hepatitis C can safely and efficiently achieve a sustained virological response (SVR) [1–4]. Although achieving SVR reduces the incidence of subsequent hepatocellular carcinoma (HCC) [5, 6], the risk of HCC occurrence may remain in some patients even after achieving SVR. In particular, patients with advanced fibrosis still have a high occurrence rate of HCC [3, 4, 7, 8]. Therefore, the guidelines of The European Association for the Study of the Liver recommend follow-up after achieving a virologic cure in patients with a METAVIR score of F3-F4 [9]. However, HCC surveillance after SVR with biannual abdominal ultrasound examinations has been reported to be less cost-effective in F3 patients [10]. Therefore, there is a need for markers that can efficiently predict the likelihood of developing HCC after SVR in patients with advanced liver fibrosis.

Fucosylation is one of the most important glycosylations involved in cancer and inflammation. We previously identified increases in fucosylated haptoglobin (Fuc-Hp) in the sera of patients with pancreatic cancer and succeeded in establishing a lectin antibody enzyme-linked immunosorbent assay (ELISA) system to measure serum Fuc-Hp levels [11]. Using this ELISA system, we found that serum Fuc-Hp levels increase as liver disease progresses from chronic hepatitis to cirrhosis and then HCC [12]. Another group also reported that the Fuc-Hp/Hp ratio was useful as a diagnostic marker for HCC, even in patients with low alpha-fetoprotein (AFP) [13]. In chronic hepatitis C patients with histologically evaluated liver fibrosis stage, Fuc-Hp levels increase with the progression of fibrosis, and patients with higher Fuc-Hp levels at the time of liver biopsy have a higher incidence of subsequent HCC [14]. However, it remains unclear whether Fuc-Hp is an independent risk factor for HCC in patients with advanced liver fibrosis. In the present study, we investigated whether Fuc-Hp could predict HCC development after SVR was achieved with DAA treatment, especially in patients with advanced liver fibrosis.

## Materials and methods

### Patients

**Fig 1:**
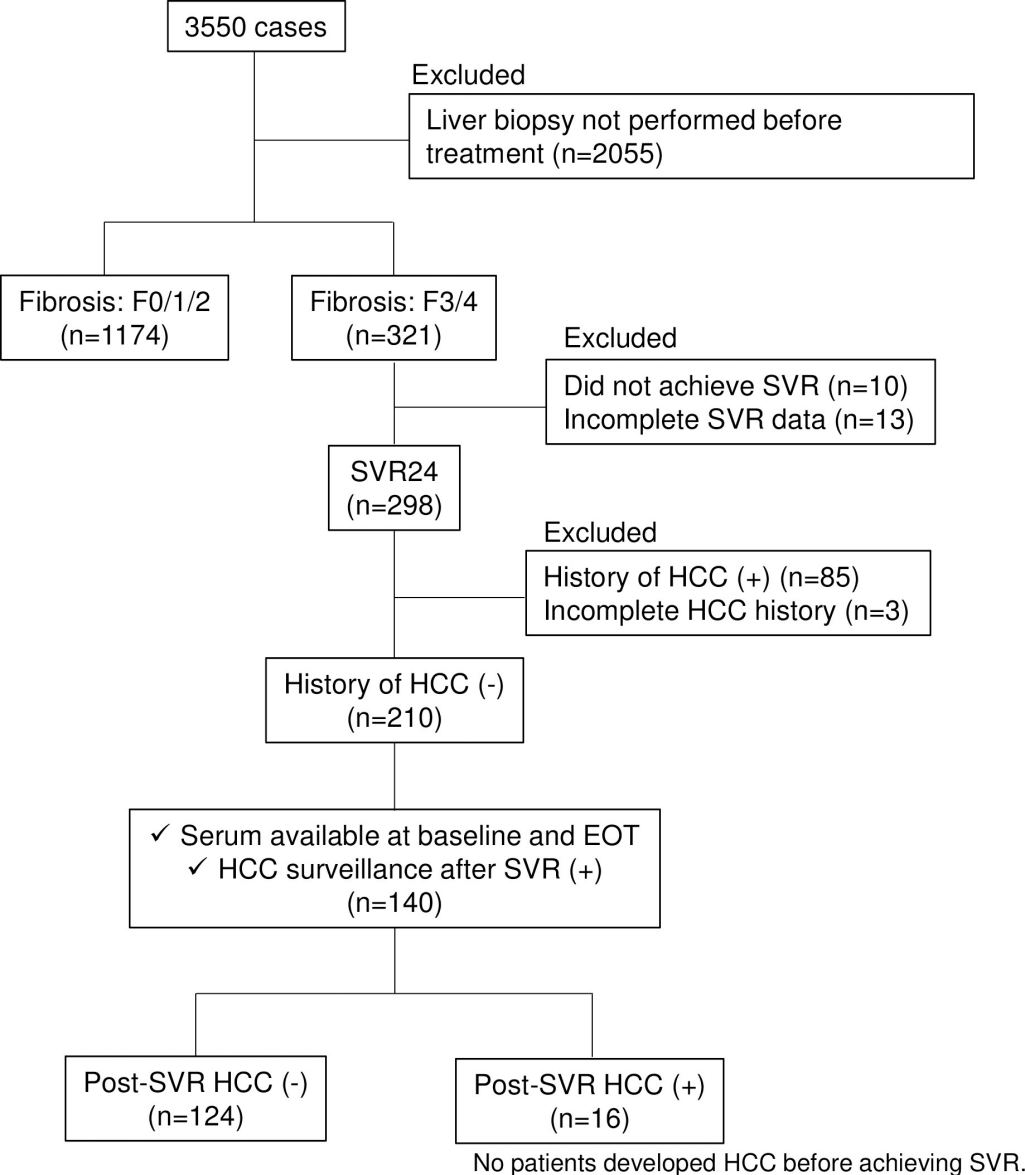
Study flowchart for enrollment in this study. Abbreviations: EOT, end of treatment; HCC, hepatocellular carcinoma; SVR, sustained virological response.

The flowchart for patient enrollment in the present study is shown in Fig 1. There were 3,550 chronic hepatitis C patients without other liver diseases who were enrolled in a prospective observational study and received DAA treatments from September 2014 to May 2020 at Osaka University Hospital and related hospitals. Among the 3,550 patients, 321 patients were diagnosed with METAVIR scores of F3 and F4 [15] by liver biopsy before DAA treatments. A SVR was defined as no detectable serum hepatitis C virus-ribonucleic acid (HCV-RNA) at 24 weeks after EOT, and 298 patients achieved SVR. We excluded patients with a history of HCC, patients whose sera from both pretreatment and EOT were not available, and patients without HCC surveillance after SVR; a total of 140 patients were included in the present study. This study was performed in accordance with the ethics guidelines outlined in the Declaration of Helsinki and was approved by the Institutional Research Board of Osaka University Hospital (No. 17032). All patients signed written informed consent forms before participating in the study. Clinical information and sera are stored anonymized, and we do not have access to information that could identify individual participants during and after data collection.

### Treatment regimens

Patients with HCV genotype 1 were treated with 24 weeks of daclatasvir and asunaprevir, 12 weeks of sofosbuvir and ledipasvir, 12 weeks of ombitasvir, paritaprevir and ritonavir, 12 weeks of elbasvir and grazoprevir or 8–12 weeks of glecaprevir and pibrentasvir. Patients with HCV genotype 2 were treated with 12 weeks of sofosbuvir and ribavirin or 8–12 weeks of glecaprevir and pibrentasvir.

### Follow-up and HCC surveillance

Prior to starting DAA treatment, all patients underwent ultrasonography, computed tomography (CT), and/or magnetic resonance imaging (MRI) to confirm the absence of HCC at that time. The patients underwent HCC surveillance using ultrasonography and/or CT/MRI at least every 6 months after starting DAA treatment. HCC was diagnosed when contrast-enhanced CT or MRI showed typical imaging findings described by the guidelines of the Japanese Society of Hepatology [16]. The observation period began at EOT. The endpoint was the date when HCC was diagnosed in patients who developed HCC or the date of the most recent follow-up imaging test in patients who did not develop HCC before 30 November 2021.

### ELISA for Fuc-Hp

The sera were stored at -80°C until analysis. The serum Fuc-Hp levels were determined using a lectin-antibody ELISA kit, as previously reported [11]. Briefly, the sera were added to an ELISA plate coated with the Fab fragment of an anti-haptoglobin polyclonal antibody to capture serum haptoglobin. Fucosylation of haptoglobin was detected using biotinylated *Aleuria aurantia* lectin.

### Statistical analysis

Fisher’s exact test was used to analyze categorical data. Statistical analysis was performed with Mann–Whitney U tests to assess differences between unpaired groups and Wilcoxon Signed-rank sum test for paired groups. Receiver operating characteristic (ROC) curve analysis was performed to assess diagnostic performance, and the area under the ROC curve (AUROC) was used to evaluate predictive power. We compared the AUROC values with the Delong test. The Youden index was used to identify the optimal cutoff points. Univariate and multivariate Cox proportional hazards models were utilized to examine the factors associated with HCC occurrence. We chose age, sex, liver function markers, liver fibrosis markers, tumor markers and factors related to diabetes as factors to perform the Cox proportional hazards model according to previous reports [17–21]. The Kaplan‒Meier method was used to assess the cumulative incidence of HCC, and the groups were compared using the log-rank test. A P value < 0.05 was considered statistically significant. All analyses were performed using Prism v.9.2.0 for Windows.

## Results

### Serum fucosylated haptoglobin levels decrease at the end of treatment

**Fig 2:**
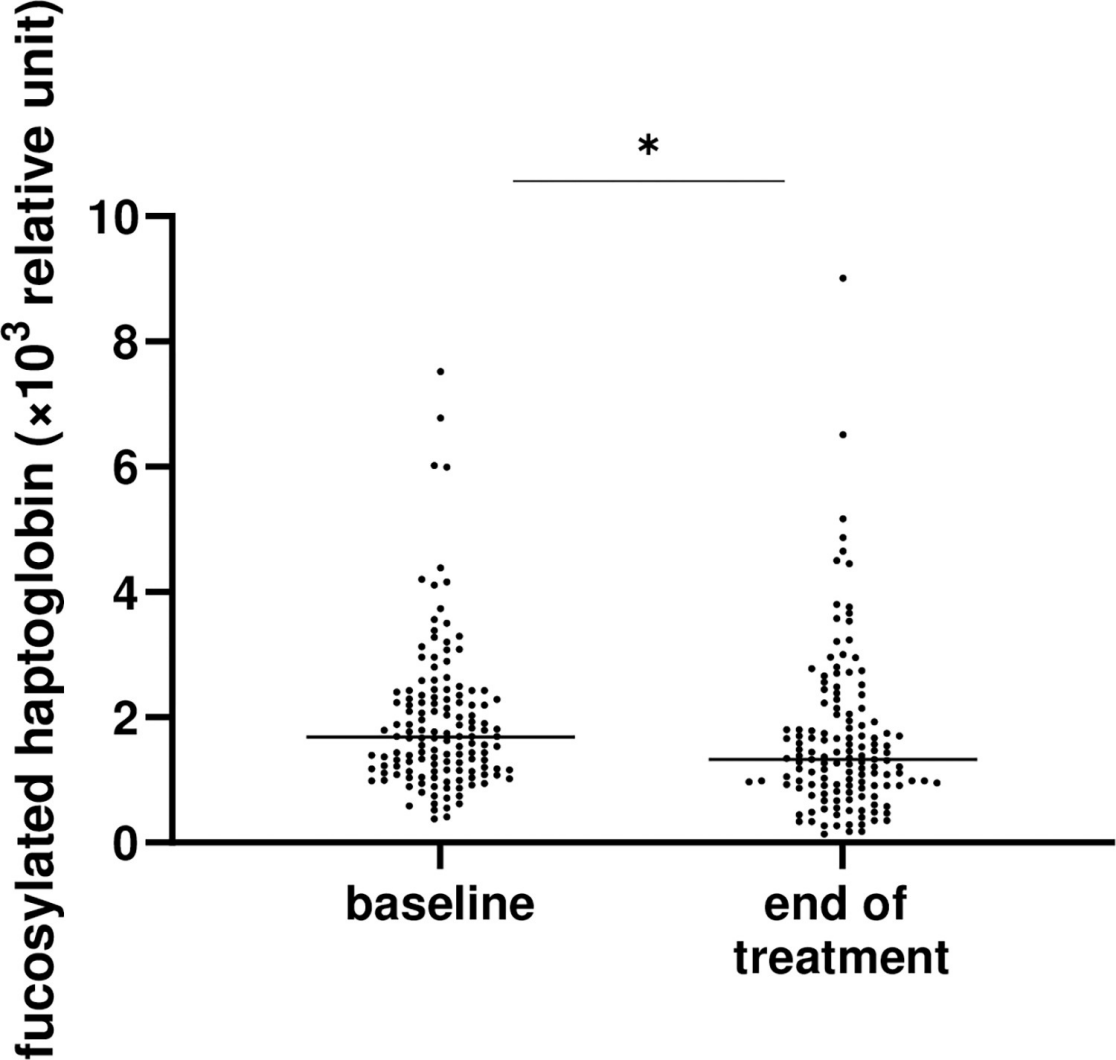
The serum levels of fucosylated haptoglobin at baseline and the end of treatment.

The characteristics of the patients at baseline are summarized in S1 Table. The median serum Fuc-Hp level at EOT was 1330 relative unit, which was significantly lower than the 1684 relative unit at baseline (Fig 2). When divided the patients into two groups according to the median Fuc-Hp level, and patients with a higher Fuc-Hp level at baseline had higher aspartate transaminase (AST), higher alanine aminotransferase (ALT), higher γ-glutamyltransferase (GGT), higher type IV collagen 7S, and higher AFP at baseline (S2 Table). Patients who were female and had higher AST, higher ALT, higher total bilirubin, higher hyaluronic acid, higher type IV collagen 7S, and higher fibrosis-4 (FIB-4) index at the end of treatment (EOT) were more likely to have higher Fuc-Hp levels at EOT (S3 Table).

### Serum fucosylated haptoglobin levels at the end of treatment associate with HCC occurrence after SVR

**Fig 3:**
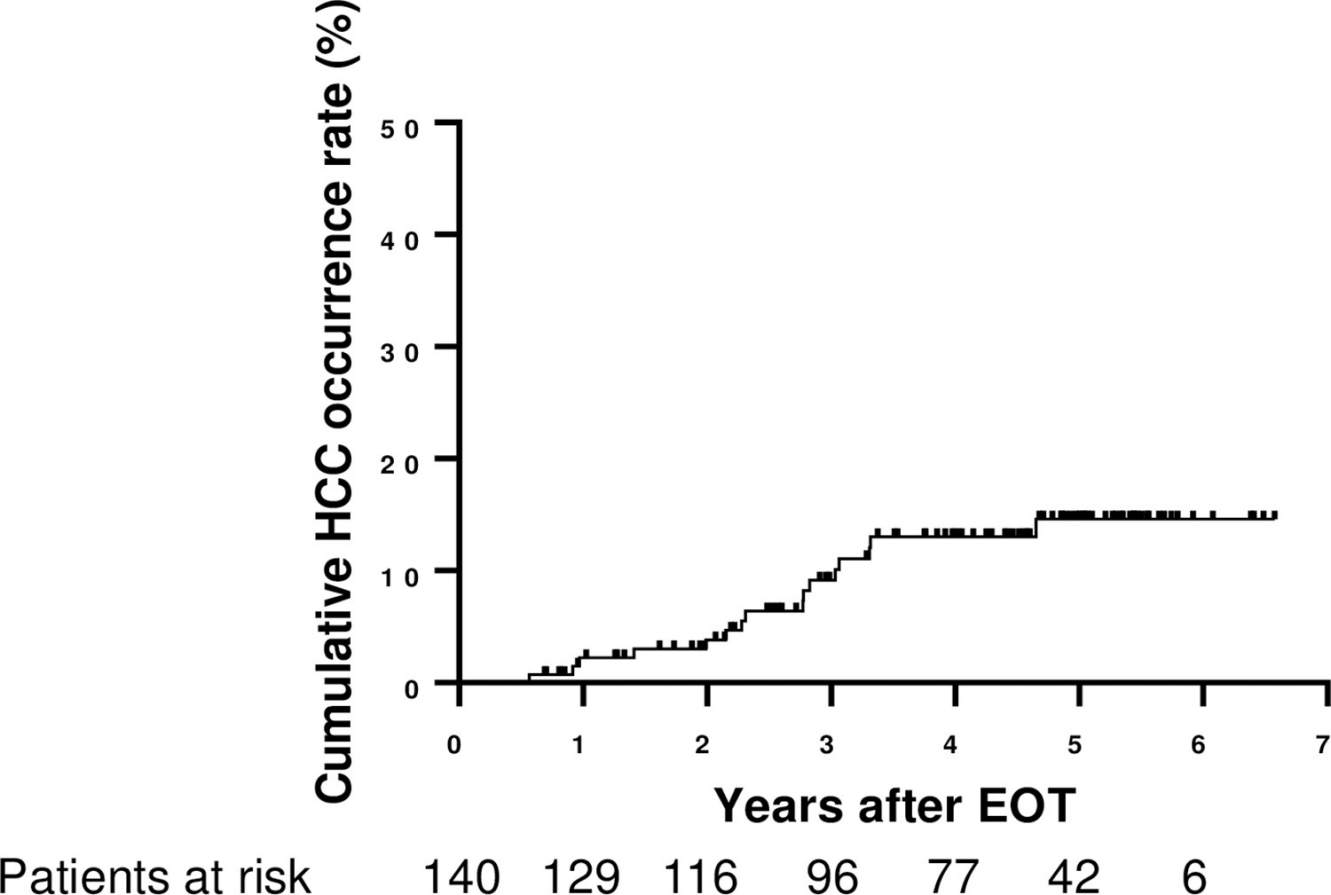
Cumulative HCC occurrence rate.

HCC occurred in 16 patients during a median follow-up of 54.4 months (Fig 3). The cumulative HCC incidence rate was 2.2% at 1 year, 9.2% at 3 years, and 14.6% at 5 years (Fig 3). We examined whether Fuc-Hp levels at baseline or EOT were risk factors for HCC occurrence after SVR was achieved with DAA treatment using Cox proportional hazards models (Table 1). In univariate analysis, high BMI, high Fuc-Hp levels and low albumin levels at EOT were significant risk factors related to HCC occurrence after HCV elimination. A multivariate analysis incorporating these three factors showed that all three factors (Fuc-Hp, p = 0.0036, BMI, p = 0.0020, albumin, p = 0.018) independently contributed to HCC occurrence after HCV elimination.

**Table 1:** The results of univariate and multivariate Cox hazard regression analyses.

		Univariate analysis			Multivariate analysis		
Factor	Category	HR	95% CI	p value	HR	95% CI	p value
Age	every 1 year	1.06	1.001–1.14	0.067			
Sex	male/female	1.1	0.41–2.94	0.86			
BMI	every 1 kg/m^2^	1.14	1.02–1.25	0.014	1.18	1.06–1.31	0.002
Fibrosis	F4/F3	2.42	0.84–6.96	0.1			
FIB4-index pre	every 1 C.O. I	1.03	0.89–1.15	0.67			
FIB4-index EOT	every 1 C.O. I	1.06	0.86–1.22	0.54			
AST pre	every 1 U/l	1.003	0.99–1.01	0.62			
AST EOT	every 1 U/l	0.99	0.95–1.01	0.54			
ALT pre	every 1 U/l	0.99	0.98–1.01	0.44			
ALT EOT	every 1 U/l	0.97	0.93–1.003	0.17			
AAR pre	every 1 C.O. I	1.98	0.75–4.02	0.10			
AAR EOT	every 1 C.O. I	1.73	0.75–3.42	0.15			
GGT pre	every 1 U/l	0.99	0.98–1.01	0.65			
GGT EOT	every 1 U/l	0.99	0.94–1.03	0.81			
HbA1c pre	every 1%	1.28	0.80–1.84	0.23			
HbA1c EOT	every 1%	1.17	0.66–1.84	0.56			
albumin pre	every 1 g/dl	0.43	0.14–1.23	0.12			
albumin EOT	every 1 g/dl	0.22	0.064–0.73	0.013	0.23	0.068–0.80	0.018
AFP pre	every 1 ng/ml	0.99	0.96–1.004	0.39			
AFP EOT	every 1 ng/ml	1.04	0.98–1.09	0.11			
DCP pre	every 1 mAU/ml	1.01	0.99–1.01	0.39			
DCP EOT	every 1 mAU/ml	1.01	0.96–1.03	0.64			
Fuc-Hp pre	every 1 ×10^3^ relative unit	1.24	0.81–1.73	0.25			
Fuc-Hp EOT	every 1 ×10^3^ relative unit	1.4	0.996–1.83	0.028	1.53	1.1–1.99	0.0036

Abbreviations: AAR, aspartate aminotransferase to alanine aminotransferase ratio; AFP, alpha-fetoprotein; AST, aspartate aminotransferase; ALT, alanine aminotransferase; BMI, body mass index; CI, confidence interval; DCP, des-γ-carboxy prothrombin; EOT, end of treatment; FIB4-index, fibrosis4-index; Fuc-Hp, fucosylated haptoglobin; GGT, gamma-glutamyl transferase; HbA1c, haemoglobin A1c; HR, hazard ratio

### Serum fucosylated haptoglobin levels combined with albumin levels and BMI stratify the risk of HCC development after SVR

**Fig 4:**
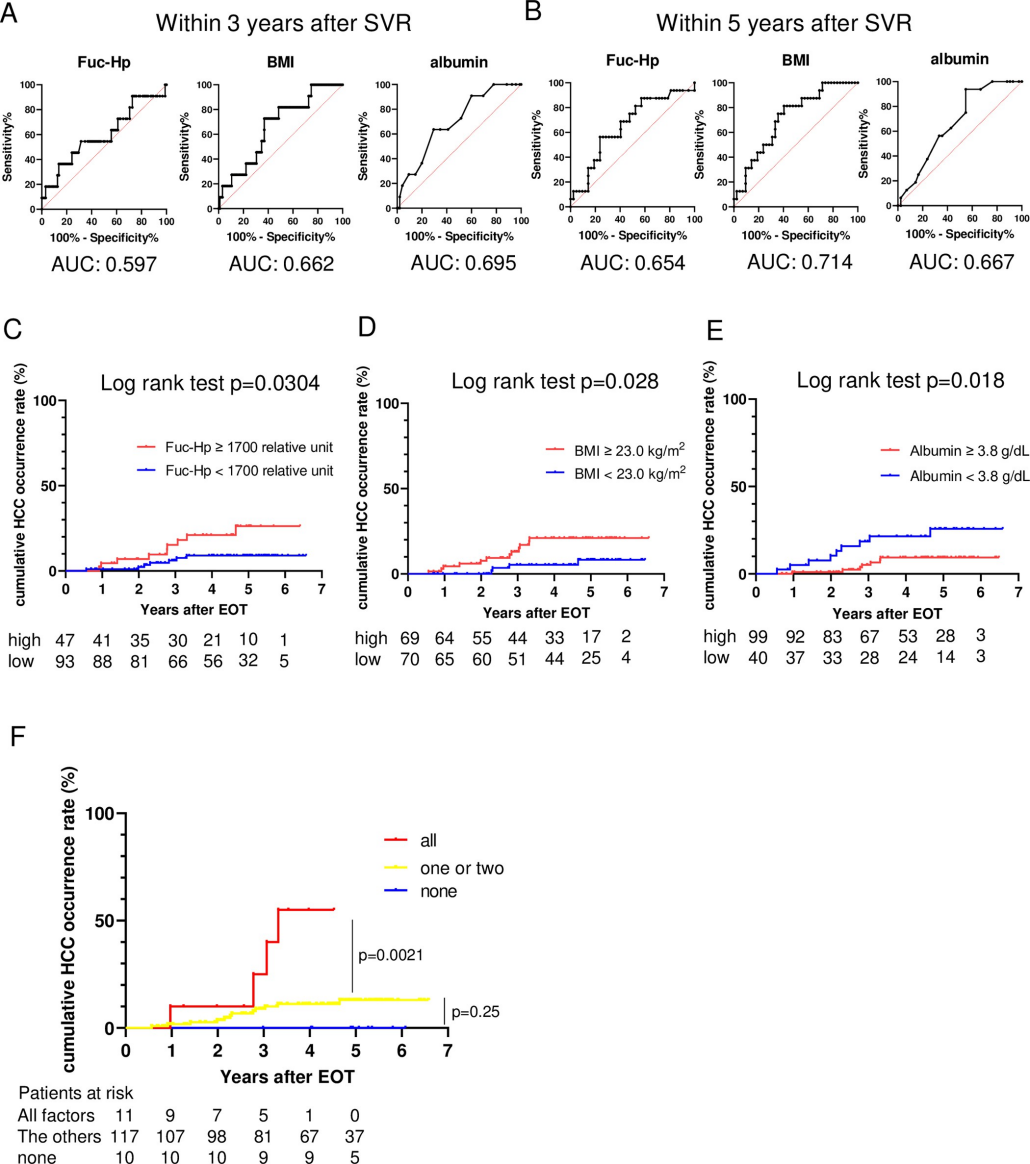
Serum fucosylated haptoglobin levels at EOT, BMI, and albumin levels at EOT stratify the risk of HCC occurrence after SVR. (A) ROC curves of Fuc-Hp, BMI, and albumin for predicting HCC occurrence within 3 years after SVR. (B) ROC curves of Fuc-Hp, BMI, and albumin for predicting HCC occurrence within 5 years after SVR. (C-E) Kaplan–Meier curves for HCC occurrence after SVR stratified according to a Fuc-Hp level of 1700 relative unit (C), BMI of 23 kg/m^2^ (D), and albumin level of 3.8 g/dl (E). (F) Kaplan–Meier curves for HCC occurrence after SVR stratified into three groups according to the number of risk factors for HCC occurrence present: all, none, and one or two factors.

The AUROCs of Fuc-Hp at EOT, BMI and albumin at EOT for predicting HCC occurrence within 3 or 5 years after SVR did not significantly differ among the three factors, according to the Delong test (Fig 4A, 4B). The optimal cutoff levels for HCC occurrence within 3 and 5 years after SVR, as calculated by the Youden index, were 1708 relative unit and 1708 relative unit for Fuc-Hp at EOT, 23.69 kg/m^2^ and 22.89 kg/m^2^ for BMI, and 3.7 g/dL and 4.1 g/dL for albumin at EOT, respectively. Based on the cutoff values calculated from the ROC curves for predicting HCC occurrence within 3 and 5 years after SVR, we set the cutoff values for Fuc-Hp, BMI, and albumin at 1700 relative unit, 23 kg/m^2^, and 3.8 g/dL, respectively. We divided patients into two groups based on each cutoff value and compared the cumulative HCC occurrence rates of each group. The cumulative HCC incidence rates in patients with Fuc-Hp levels at EOT ≥ 1700 relative unit and < 1700 relative unit were 15.3% and 6.2% at 3 years and 26.3% and 9.1% at 5 years, respectively (Fig 4C). The cumulative HCC incidence rates in patients with a BMI ≥ 23 kg/m^2^ and < 23 kg/m^2^ were 13.1% and 5.4% at 3 years and 21.1% and 8.3% at 5 years, respectively (Fig 4D). The cumulative HCC incidence rates in patients with albumin levels ≥ 3.8 g/dL and < 3.8 g/dL were 5.1% and 18.6% at 3 years and 9.4% and 25.8% at 5 years, respectively (Fig 4E). Among patients with advanced liver fibrosis, those with Fuc-Hp ≥ 1700 relative unit, BMI ≥ 23 kg/m^2^, and albumin < 3.8 g/dL had a significantly higher incidence of HCC than patients not meeting these cutoffs, with a 25% incidence within 3 years and a 55% incidence within 4 years (Fig 4F). In contrast, none of the patients with Fuc-Hp < 1700 relative unit, BMI < 23 kg/m^2^, and albumin ≥ 3.8 g/dL developed HCC during the median 5-year observation period (Fig 4F).

## Discussion

In the present study, we identified that serum Fuc-Hp levels decreased after HCV elimination and that a high Fuc-Hp level at EOT was an independent factor contributing to the development of HCC after SVR in F3 and F4 patients. Based on Fuc-Hp levels at EOT, BMI, and albumin levels at EOT, we were able to stratify the risk of HCC occurrence after SVR among patients with advanced liver fibrosis.

Glycosylation is one of the most important posttranslational modifications of proteins and lipids [22]. There are two major types of glycosylation in glycoproteins, N-glycosylation and O-glycosylation [23]. Fucosylation is a type of glycan change in N-glycans and increases in states of inflammation [24]. Inflammatory stimuli, such as IL6, increase the expression of fucosylation regulatory genes [25]. It has been demonstrated that N-glycans of glycoproteins such as haptoglobin, α1-antitrypsin, α-fetoprotein, immunoglobulins, and others change during inflammation caused by hepatitis virus [26]. An in vitro examination showed that fucosylation levels are significantly increased by HCV infection in human hepatocytes [27]. Furthermore, haptoglobin is an acute phase protein mainly produced in the liver and increases in response to inflammatory stimuli [28]. Chronic HCV infection is accompanied by an increase in acute phase proteins, which are mostly glycoproteins [29]. Among chronic hepatitis C patients with pathological activity, patients with METAVIR scores of A1-3 have higher serum Fuc-Hp levels than those with A0 [12]. However, changes in serum Fuc-Hp levels after HCV elimination have not yet been reported. In the present study, we revealed that serum Fuc-Hp levels decreased after HCV elimination. It is speculated that the elimination of HCV reduces the inflammatory stimulus, resulting in a reduction in the amount of haptoglobin and its degree of fucosylation, both of which contribute to decreased serum Fuc-Hp levels after HCV elimination.

In the present study, a high Fuc-Hp level at EOT was an independent risk factor for the development of HCC after SVR. Altered glycosylation in serum proteins is a frequent event during tumor development and progression and thus served as a promising biomarker [30]. Fucosylation status is elevated in cancer tissue due to the induction of several glycosyltransferases, GDP-fucose production, and upregulation of the GDP-fucose transporter [31]. In addition to the increased fucosylation status, cell polarity contributes to serum levels of Fuc-Hp [32, 33]. Namely, fucosylated glycoproteins produced intracellularly are secreted into bile in normal hepatocytes [32]. However, in cells that have lost polarity, such as hepatoma cells, fucosylated glycoproteins are also secreted into the blood, resulting in increased serum concentrations [33]. Collectively, the presence of malignant transformed cells with lost polarity increases serum Fuc-Hp levels via both increased fucosylation status and increased secretion of fucosylated proteins into the serum, which may explain why high Fuc-Hp levels after DAA treatment were a risk factor for HCC development.

Previous studies have reported that AFP [34], the FIB-4 index [17], Mac 2-binding protein glycan isomer (M2BPGi) [35], and GGT [21] are risk factors for the development of HCC after SVR in patients with chronic hepatitis C. Elevated levels of AFP, the FIB-4 index, and M2BPGi are associated with advanced liver fibrosis, which is a well-known HCC risk factor [36–38]; however, it remains unclear how strong these markers are as risk factors for HCC occurrence after SVR among patients with advanced fibrosis. In the present study, only patients with advanced liver fibrosis diagnosed on liver biopsy were included. The median baseline levels of AFP and the FIB-4 index in the present study were 10 ng/ml and 4.61, respectively (S1 Table), higher than those reported in previous studies [17, 34, 39]. On the other hand, GGT has been reported to be a risk factor for HCC development in only noncirrhotic patients [21]. In the present study, Fuc-Hp but not AFP, the FIB-4 index or GGT was shown to be an independent risk factor for the development of HCC in patients with advanced liver fibrosis, suggesting that Fuc-Hp may be more useful than these other markers in stratifying the risk of HCC development in patients with advanced liver fibrosis.

On the other hand, baseline Fuc-Hp was not associated with the development of HCC in the present study. One drawback of Fuc-Hp is that it cannot stratify the risk of developing HCC after SVR before the initiation of antiviral therapy. In addition, one technical note is that hemolysis must be avoided when measuring Fuc-Hp, since hemoglobin contamination has been shown to reduce the measured concentration of Fuc-Hp [11].

Since clinical factors at EOT, such as AFP levels, have been reported as risk factors for the development of HCC after SVR [40, 41], and the present study included factors at EOT as well as at baseline in the analysis. High BMI and low albumin at EOT were also found to be independent risk factors for the development of HCC after SVR. Obesity, a main component of metabolic syndrome, causes remodeling of adipose tissue. Obesity potentially accelerates hepatocarcinogenesis by inducing increased secretion of proinflammatory adipokines and decreased secretion of anti-inflammatory adipokines [42, 43]. Obesity has been reported to be a risk factor for HCC, including in studies on hepatitis C [44, 45]. Albumin is a factor included in the albumin-bilirubin (ALBI) score and the Child‒Pugh classification, which reflect liver function [46, 47]. Low serum albumin levels when achieving SVR are a risk factor for carcinogenesis after SVR [48, 49]. Although serum albumin levels increase with HCV elimination [50–52], patients with inadequate improvement in liver function may still develop HCC after SVR. In the present study, patients with higher BMI, higher Fuc-Hp at EOT, and lower albumin at EOT had a higher incidence of HCC. On the other hand, although observed in a small number of patients, those with lower BMI, lower Fuc-Hp, and higher albumin did not develop HCC during the observation period of the present study. The combination of Fuc-Hp levels suggesting hepatocyte degeneration, albumin levels reflecting liver function, and obesity associated with carcinogenesis may be useful for further stratifying the risk of carcinogenesis among patients with advanced fibrosis.

In the current study, patients with advanced liver fibrosis were selected based on histological evaluation by liver biopsy, the gold standard for diagnosis of liver fibrosis. Since liver biopsy is an invasive procedure, many patients do not undergo it prior to antiviral therapy, which contributed to the small number of cases in the present study.

In conclusion, serum Fuc-Hp levels decrease after HCV elimination. High serum Fuc-Hp levels at EOT is a risk factor for the development of HCC after SVR. Among patients with advanced liver fibrosis, patients who have high Fuc-Hp levels at EOT, high BMI, and low albumin levels at EOT should be carefully monitored for HCC occurrence after HCV elimination.

## Supporting information

S1 Checklist STROBE statement—checklist of items that should be included in reports of observational studies. (DOCX)

S1 Table Patient characteristics at baseline (median (IQR)). (DOCX)

S2 Table Characteristics of patients with high Fuc-Hp and low Fuc-Hp at baseline. (DOCX)

S3 Table Characteristics of patients with high Fuc-Hp and low Fuc-Hp at the end of treatment. (DOCX)

## Abbreviations

AFP: alpha-fetoprotein
ALBI: albumin-bilirubin
ALT: alanine aminotransferase
AST: aspartate transaminase
AUROC: area under the receiver operating characteristic curve
BMI: body mass index
CT: computed tomography
DAA: direct-acting antiviral
ELISA: enzyme-linked immunosorbent assay
EOT: end of treatment
FIB-4 index: fibrosis-4 index
Fuc-Hp: fucosylated haptoglobin
GGT: γ-glutamyltransferase
HCC: hepatocellular carcinoma
HCV-RNA: hepatitis C virus-ribonucleic acid
M2BPGi: Mac 2-binding protein glycan isomer
MRI: magnetic resonance imaging
ROC: receiver operating characteristic
SVR: sustained virological response

## References

[1] Maan R, van Tilborg M, Deterding K, Ramji A, van der Meer AJ, Wong F, et al. Safety and Effectiveness of Direct-Acting Antiviral Agents for Treatment of Patients With Chronic Hepatitis C Virus Infection and Cirrhosis. Clin Gastroenterol Hepatol. 2016;14(12):1821–30.e6. doi: 10.1016/j.cgh.2016.07.001 27404965

[2] Hézode C. Treatment of hepatitis C: Results in real life. Liver Int. 2018;38 Suppl 1:21–7. doi: 10.1111/liv.13638 29427481

[3] Yoshiji H, Nagoshi S, Akahane T, Asaoka Y, Ueno Y, Ogawa K, et al. Evidence-based clinical practice guidelines for liver cirrhosis 2020. Hepatol Res. 2021;51(7):725–49. doi: 10.1111/hepr.13678 34228859

[4] Yoshiji H, Nagoshi S, Akahane T, Asaoka Y, Ueno Y, Ogawa K, et al. Evidence-based clinical practice guidelines for Liver Cirrhosis 2020. J Gastroenterol. 2021;56(7):593–619. doi: 10.1007/s00535-021-01788-x 34231046 PMC8280040

[5] Backus LI, Belperio PS, Shahoumian TA, Mole LA. Impact of Sustained Virologic Response with Direct-Acting Antiviral Treatment on Mortality in Patients with Advanced Liver Disease. Hepatology. 2019;69(2):487–97. doi: 10.1002/hep.29408 28749564

[6] Calvaruso V, Cabibbo G, Cacciola I, Petta S, Madonia S, Bellia A, et al. Incidence of Hepatocellular Carcinoma in Patients With HCV-Associated Cirrhosis Treated With Direct-Acting Antiviral Agents. Gastroenterology. 2018;155(2):411–21.e4. doi: 10.1053/j.gastro.2018.04.008 29655836

[7] Kanwal F, Kramer J, Asch SM, Chayanupatkul M, Cao Y, El-Serag HB. Risk of Hepatocellular Cancer in HCV Patients Treated With Direct-Acting Antiviral Agents. Gastroenterology. 2017;153(4):996–1005.e1. doi: 10.1053/j.gastro.2017.06.012 28642197

[8] Ioannou GN, Beste LA, Green PK, Singal AG, Tapper EB, Waljee AK, et al. Increased Risk for Hepatocellular Carcinoma Persists Up to 10 Years After HCV Eradication in Patients With Baseline Cirrhosis or High FIB-4 Scores. Gastroenterology. 2019;157(5):1264–78.e4. 31356807 10.1053/j.gastro.2019.07.033 PMC6815714

[9] easloffice@easloffice.eu EAftSotLEa, Chair: CPGP, representative: EGB, members: P. EASL recommendations on treatment of hepatitis C: Final update of the series. J Hepatol. 2020;73(5):1170–218. 32956768 10.1016/j.jhep.2020.08.018

[10] Farhang Zangneh H, Wong WWL, Sander B, Bell CM, Mumtaz K, Kowgier M, et al. Cost Effectiveness of Hepatocellular Carcinoma Surveillance After a Sustained Virologic Response to Therapy in Patients With Hepatitis C Virus Infection and Advanced Fibrosis. Clin Gastroenterol Hepatol. 2019;17(9):1840–9.e16. 30580095 10.1016/j.cgh.2018.12.018

[11] Kamada Y, Kinoshita N, Tsuchiya Y, Kobayashi K, Fujii H, Terao N, et al. Reevaluation of a lectin antibody ELISA kit for measuring fucosylated haptoglobin in various conditions. Clin Chim Acta. 2013;417:48–53. doi: 10.1016/j.cca.2012.12.014 23262369

[12] Asazawa H, Kamada Y, Takeda Y, Takamatsu S, Shinzaki S, Kim Y, et al. Serum fucosylated haptoglobin in chronic liver diseases as a potential biomarker of hepatocellular carcinoma development. Clin Chem Lab Med. 2015;53(1):95–102. doi: 10.1515/cclm-2014-0427 25060348

[13] Shang S, Li W, Qin X, Zhang S, Liu Y. Aided Diagnosis of Hepatocellular Carcinoma Using Serum Fucosylated Haptoglobin Ratios. J Cancer. 2017;8(5):887–93. doi: 10.7150/jca.17747 28382152 PMC5381178

[14] Tawara S, Tatsumi T, Iio S, Kobayashi I, Shigekawa M, Hikita H, et al. Evaluation of Fucosylated Haptoglobin and Mac-2 Binding Protein as Serum Biomarkers to Estimate Liver Fibrosis in Patients with Chronic Hepatitis C. PLoS One. 2016;11(3):e0151828. doi: 10.1371/journal.pone.0151828 27002630 PMC4803196

[15] Intraobserver and interobserver variations in liver biopsy interpretation in patients with chronic hepatitis C. The French METAVIR Cooperative Study Group. Hepatology. 1994;20(1):15–20. 8020885

[16] Kokudo N, Takemura N, Hasegawa K, Takayama T, Kubo S, Shimada M, et al. Clinical practice guidelines for hepatocellular carcinoma: The Japan Society of Hepatology 2017 (4th JSH-HCC guidelines) 2019 update. Hepatol Res. 2019;49(10):1109–13. doi: 10.1111/hepr.13411 31336394

[17] Tahata Y, Sakamori R, Yamada R, Kodama T, Hikita H, Hagiwara H, et al. Prediction model for hepatocellular carcinoma occurrence in patients with hepatitis C in the era of direct-acting anti-virals. Aliment Pharmacol Ther. 2021;54(10):1340–9. doi: 10.1111/apt.16632 34618934

[18] Degasperi E, D’Ambrosio R, Iavarone M, Sangiovanni A, Aghemo A, Soffredini R, et al. Factors Associated With Increased Risk of De Novo or Recurrent Hepatocellular Carcinoma in Patients With Cirrhosis Treated With Direct-Acting Antivirals for HCV Infection. Clin Gastroenterol Hepatol. 2019;17(6):1183–91.e7. doi: 10.1016/j.cgh.2018.10.038 30613002

[19] Tani J, Morishita A, Sakamoto T, Takuma K, Nakahara M, Fujita K, et al. Simple scoring system for prediction of hepatocellular carcinoma occurrence after hepatitis C virus eradication by direct-acting antiviral treatment: All Kagawa Liver Disease Group Study. Oncol Lett. 2020;19(3):2205–12. doi: 10.3892/ol.2020.11341 32194718 PMC7038998

[20] Ioannou GN, Green PK, Beste LA, Mun EJ, Kerr KF, Berry K. Development of models estimating the risk of hepatocellular carcinoma after antiviral treatment for hepatitis C. J Hepatol. 2018;69(5):1088–98. doi: 10.1016/j.jhep.2018.07.024 30138686 PMC6201746

[21] Huang CF, Yeh ML, Tsai PC, Hsieh MH, Yang HL, Hsieh MY, et al. Baseline gamma-glutamyl transferase levels strongly correlate with hepatocellular carcinoma development in non-cirrhotic patients with successful hepatitis C virus eradication. J Hepatol. 2014;61(1):67–74. doi: 10.1016/j.jhep.2014.02.022 24613362

[22] Shang S, Qin X, Li W, Zhang S, Liu Y. ELISA index of serum fucosylated haptoglobin for diagnosis of HCC using the normal and reverse AAL ELISA. Discov Med. 2016;21(113):15–23. 26896598

[23] Miyoshi E, Kamada Y, Suzuki T. Functional glycomics: Application to medical science and hepatology. Hepatol Res. 2020;50(2):153–64. doi: 10.1111/hepr.13459 31750967

[24] Okuyama N, Ide Y, Nakano M, Nakagawa T, Yamanaka K, Moriwaki K, et al. Fucosylated haptoglobin is a novel marker for pancreatic cancer: a detailed analysis of the oligosaccharide structure and a possible mechanism for fucosylation. Int J Cancer. 2006;118(11):2803–8. doi: 10.1002/ijc.21728 16385567

[25] Narisada M, Kawamoto S, Kuwamoto K, Moriwaki K, Nakagawa T, Matsumoto H, et al. Identification of an inducible factor secreted by pancreatic cancer cell lines that stimulates the production of fucosylated haptoglobin in hepatoma cells. Biochem Biophys Res Commun. 2008;377(3):792–6. doi: 10.1016/j.bbrc.2008.10.061 18951869

[26] Gornik O, Lauc G. Glycosylation of serum proteins in inflammatory diseases. Dis Markers. 2008;25(4–5):267–78. doi: 10.1155/2008/493289 19126970 PMC3827815

[27] Xiang T, Yang G, Liu X, Zhou Y, Fu Z, Lu F, et al. Alteration of N-glycan expression profile and glycan pattern of glycoproteins in human hepatoma cells after HCV infection. Biochim Biophys Acta Gen Subj. 2017;1861(5 Pt A):1036–45. doi: 10.1016/j.bbagen.2017.02.014 28229927

[28] Qin X, Guo Y, Du H, Zhong Y, Zhang J, Li X, et al. Comparative Analysis for Glycopatterns and Complex-Type. Front Physiol. 2017;8:596. 28871230 10.3389/fphys.2017.00596 PMC5566988

[29] Arnold JN, Saldova R, Hamid UM, Rudd PM. Evaluation of the serum N-linked glycome for the diagnosis of cancer and chronic inflammation. Proteomics. 2008;8(16):3284–93. doi: 10.1002/pmic.200800163 18646009

[30] Reis CA, Osorio H, Silva L, Gomes C, David L. Alterations in glycosylation as biomarkers for cancer detection. J Clin Pathol. 2010;63(4):322–9. doi: 10.1136/jcp.2009.071035 20354203

[31] Miyoshi E, Moriwaki K, Terao N, Tan CC, Terao M, Nakagawa T, et al. Fucosylation is a promising target for cancer diagnosis and therapy. Biomolecules. 2012;2(1):34–45. doi: 10.3390/biom2010034 24970126 PMC4030867

[32] Nakagawa T, Uozumi N, Nakano M, Mizuno-Horikawa Y, Okuyama N, Taguchi T, et al. Fucosylation of N-glycans regulates the secretion of hepatic glycoproteins into bile ducts. J Biol Chem. 2006;281(40):29797–806. doi: 10.1074/jbc.M605697200 16899455

[33] Nakagawa T, Moriwaki K, Terao N, Miyamoto Y, Kamada Y, Miyoshi E. Analysis of polarized secretion of fucosylated alpha-fetoprotein in HepG2 cells. J Proteome Res. 2012;11(5):2798–806. doi: 10.1021/pr201154k 22483194

[34] Tanaka Y, Ogawa E, Huang CF, Toyoda H, Jun DW, Tseng CH, et al. HCC risk post-SVR with DAAs in East Asians: findings from the REAL-C cohort. Hepatol Int. 2020;14(6):1023–33. doi: 10.1007/s12072-020-10105-2 33277685

[35] Nagata H, Nakagawa M, Asahina Y, Sato A, Asano Y, Tsunoda T, et al. Effect of interferon-based and -free therapy on early occurrence and recurrence of hepatocellular carcinoma in chronic hepatitis C. J Hepatol. 2017;67(5):933–9. doi: 10.1016/j.jhep.2017.05.028 28627363

[36] Hu KQ, Kyulo NL, Lim N, Elhazin B, Hillebrand DJ, Bock T. Clinical significance of elevated alpha-fetoprotein (AFP) in patients with chronic hepatitis C, but not hepatocellular carcinoma. Am J Gastroenterol. 2004;99(5):860–5. doi: 10.1111/j.1572-0241.2004.04152.x 15128351

[37] Vallet-Pichard A, Mallet V, Nalpas B, Verkarre V, Nalpas A, Dhalluin-Venier V, et al. FIB-4: an inexpensive and accurate marker of fibrosis in HCV infection. comparison with liver biopsy and fibrotest. Hepatology. 2007;46(1):32–6. doi: 10.1002/hep.21669 17567829

[38] Kuno A, Ikehara Y, Tanaka Y, Ito K, Matsuda A, Sekiya S, et al. A serum "sweet-doughnut" protein facilitates fibrosis evaluation and therapy assessment in patients with viral hepatitis. Sci Rep. 2013;3:1065. doi: 10.1038/srep01065 23323209 PMC3545220

[39] Tamaki N, Kurosaki M, Yasui Y, Mori N, Tsuji K, Hasebe C, et al. Change in Fibrosis 4 Index as Predictor of High Risk of Incident Hepatocellular Carcinoma After Eradication of Hepatitis C Virus. Clin Infect Dis. 2021;73(9):e3349–e54. doi: 10.1093/cid/ciaa1307 33544129 PMC8824825

[40] Masetti C, Lionetti R, Lupo M, Siciliano M, Giannelli V, Ponziani FR, et al. Lack of reduction in serum alpha-fetoprotein during treatment with direct antiviral agents predicts hepatocellular carcinoma development in a large cohort of patients with hepatitis C virus-related cirrhosis. J Viral Hepat. 2018;25(12):1493–500. doi: 10.1111/jvh.12982 30112854

[41] Mawatari S, Kumagai K, Oda K, Tabu K, Ijuin S, Fujisaki K, et al. Features of patients who developed hepatocellular carcinoma after direct-acting antiviral treatment for hepatitis C Virus. PLoS One. 2022;17(1):e0262267. doi: 10.1371/journal.pone.0262267 35020772 PMC8754290

[42] Park EJ, Lee JH, Yu GY, He G, Ali SR, Holzer RG, et al. Dietary and genetic obesity promote liver inflammation and tumorigenesis by enhancing IL-6 and TNF expression. Cell. 2010;140(2):197–208. doi: 10.1016/j.cell.2009.12.052 20141834 PMC2836922

[43] Dalamaga M, Diakopoulos KN, Mantzoros CS. The role of adiponectin in cancer: a review of current evidence. Endocr Rev. 2012;33(4):547–94. doi: 10.1210/er.2011-1015 22547160 PMC3410224

[44] Ohki T, Tateishi R, Sato T, Masuzaki R, Imamura J, Goto T, et al. Obesity is an independent risk factor for hepatocellular carcinoma development in chronic hepatitis C patients. Clin Gastroenterol Hepatol. 2008;6(4):459–64. doi: 10.1016/j.cgh.2008.02.012 18387499

[45] Tateishi R, Matsumura T, Okanoue T, Shima T, Uchino K, Fujiwara N, et al. Hepatocellular carcinoma development in diabetic patients: a nationwide survey in Japan. J Gastroenterol. 2021;56(3):261–73. doi: 10.1007/s00535-020-01754-z 33427937 PMC7932951

[46] Johnson PJ, Berhane S, Kagebayashi C, Satomura S, Teng M, Reeves HL, et al. Assessment of liver function in patients with hepatocellular carcinoma: a new evidence-based approach-the ALBI grade. J Clin Oncol. 2015;33(6):550–8. doi: 10.1200/JCO.2014.57.9151 25512453 PMC4322258

[47] Pugh RN, Murray-Lyon IM, Dawson JL, Pietroni MC, Williams R. Transection of the oesophagus for bleeding oesophageal varices. Br J Surg. 1973;60(8):646–9. doi: 10.1002/bjs.1800600817 4541913

[48] Semmler G, Meyer EL, Kozbial K, Schwabl P, Hametner-Schreil S, Zanetto A, et al. HCC risk stratification after cure of hepatitis C in patients with compensated advanced chronic liver disease. J Hepatol. 2022;76(4):812–21. doi: 10.1016/j.jhep.2021.11.025 34871626

[49] Pons M, Rodríguez-Tajes S, Esteban JI, Mariño Z, Vargas V, Lens S, et al. Non-invasive prediction of liver-related events in patients with HCV-associated compensated advanced chronic liver disease after oral antivirals. J Hepatol. 2020;72(3):472–80. doi: 10.1016/j.jhep.2019.10.005 31629779

[50] Martínez Herreros Á, Sangro B, García Rodriguez A, Pérez Grijalba V. Analysis of the albumin-bilirubin score as an indicator of improved liver function among hepatitis C virus patients with sustained viral response after direct-acting antiviral therapy. JGH Open. 2022;6(7):496–502. doi: 10.1002/jgh3.12779 35822123 PMC9260218

[51] Atsukawa M, Tsubota A, Kondo C, Toyoda H, Nakamuta M, Takaguchi K, et al. Time-course changes in liver functional reserve after successful sofosbuvir/velpatasvir treatment in patients with decompensated cirrhosis. Hepatol Res. 2022;52(3):235–46. doi: 10.1111/hepr.13739 34861090

[52] Tahata Y, Hikita H, Mochida S, Kawada N, Enomoto N, Ido A, et al. Sofosbuvir plus velpatasvir treatment for hepatitis C virus in patients with decompensated cirrhosis: a Japanese real-world multicenter study. J Gastroenterol. 2021;56(1):67–77. doi: 10.1007/s00535-020-01733-4 33001338

