# Supplementary material for: Fucosylated haptoglobin is a novel predictive marker of hepatocellular carcinoma after hepatitis C virus elimination in patients with advanced liver fibrosis
Source: PLoS One. 2022 Dec 21;17(12):e0279416. doi: 10.1371/journal.pone.0279416 (PMC9770342; doi:10.1371/journal.pone.0279416)
Supplement: S1 Table — (DOCX) [file pone.0279416.s002.docx]

S1 Table. Patients’ characteristics at baseline (median (IQR))

| Factor | Baseline |
| --- | --- |
| Age (years) | 67 (62-75) |
| Sex: male/female (% male) | 61/79 (43.6%) |
| BMI (kg/m^2^) | 22.9 (20.5-24.8) |
| Fibrosis: F3/F4 | 73/67 |
| DCV/ASV, SOF/LDV, SOF/RBV, OBV/PTV/r, EBR/GZR, GLE/PIB | 18/93/17/4/4/4 |
| HCV-RNA (LogIU/ml) | 6.1 (5.6-6.5) |
| Platelets (×10^4^/μl) | 11.8 (8.8-15) |
| AST (U/l) | 54 (42-81) |
| ALT (U/l) | 49 (34-81) |
| GGT (U/l) | 43 (27-70) |
| Total bilirubin (mg/dl) | 0.7 (0.6-0.9) |
| eGFR | 72.7 (60.2-80.7) |
| FBG (mg/dL) | 105 (94-131) |
| HbA1c (%) | 5.5 (5.2-6.0) |
| Albumin (g/dl) | 3.7 (3.5-4.1) |
| PT (%) | 83 (75-90) |
| Hyaluronic acid (ng/ml) | 255 (129-466) |
| Type 4 collagen 7S (ng/ml) | 8.0 (6.3-10.0) |
| AFP (ng/ml) | 10 (5-20) |
| DCP (mAU/ml) | 19 (15-25) |
| FIB-4 index | 4.61 (2.99-7.43) |
| ALBI score | -2.48 (-2.78— -2.17) |

Abbreviations: AFP, alpha-fetoprotein; ALBI, albumin-bilirubin; ALT, alanine aminotransferase; AST, aspartate transaminase; ASV, asunaprevir; BMI, body mass index; DCP, des-γ- carboxy prothrombin; DCV, daclatasvir; EBR, elbasvir; eGFR, estimated glomerular filtration rate; FBG, fasting blood glucose; FIB-4 index, fibrosis-4 index; GGT, γ- glutamyltransferase; GLE, glecaprevir; GZR, grazoprevir; HbA1c, hemoglobin A1c; HCV-RNA, hepatitis C virus-ribonucleic acid; LDV, ledipasvir; OBV, ombitasvir; PIB, pibrentasvir; PT, prothrombin time; PTV, paritaprevir; r, ritonavir; RBV, ribavirin; SOF, sofosbuvir
