# Supplementary material for: Fucosylated haptoglobin is a novel predictive marker of hepatocellular carcinoma after hepatitis C virus elimination in patients with advanced liver fibrosis
Source: PLoS One. 2022 Dec 21;17(12):e0279416. doi: 10.1371/journal.pone.0279416 (PMC9770342; doi:10.1371/journal.pone.0279416)
Supplement: S2 Table — (DOCX) [file pone.0279416.s003.docx]

S2 Table. Characteristics of patients with high Fuc-Hp and low Fuc-Hp at baseline

| Factor | Fuc-Hp ≤1684 relative unit (n=70) | Fuc-Hp >1684 relative unit (n=70) | p value |
| --- | --- | --- | --- |
| Age (years) | 67 (63-73) | 68 (61-76) | 0.79 |
| Sex: male/female (% male) | 33/37 (47.1%) | 28/42 (40%) | 0.5 |
| BMI (kg/m^2^) | 23.5 (20.6-25.1) | 22.8 (20.4-24.2) | 0.17 |
| Fibrosis: F3/F4 | 38/32 | 35/35 | 0.74 |
| HCV-RNA (LogIU/ml) | 6.2 (5.7-6.5) | 6.0 (5.5-6.4) | 0.23 |
| Platelets (×10^4^/μl) | 12.0 (8.5-15) | 11.5 (8.9-14.9) | 0.85 |
| AST (U/l) | 48 (36-68) | 59 (47-92) | 0.0031 |
| ALT (U/l) | 42 (28-64) | 59 (40-89) | 0.0007 |
| GGT (U/l) | 36 (22-59) | 47 (32-81) | 0.0039 |
| Total bilirubin (mg/dl) | 0.7 (0.6-0.9) | 0.7 (0.6-1.0) | 0.18 |
| eGFR | 70.7 (61.1-80.7) | 73.1 (57.1-80.1) | 0.97 |
| FBG (mg/dL) | 104 (94-130) | 106 (94-134) | 0.49 |
| HbA1c (%) | 5.5 (5.2-6.1) | 5.5 (5.3-6.0) | 0.85 |
| Albumin (g/dl) | 3.8 (3.5-4.1) | 3.7 (3.4-4.1) | 0.32 |
| PT (%) | 84 (76-90) | 82 (75-91) | 0.76 |
| Hyaluronic acid (ng/ml) | 231 (99-417) | 272 (144-489) | 0.23 |
| Type 4 collagen 7S (ng/ml) | 7.2 (5.9-8.7) | 8.5 (7.0-10.0) | 0.0072 |
| AFP (ng/ml) | 7 (4-15) | 12 (7-24) | 0.002 |
| DCP (mAU/ml) | 19 (15-24) | 20 (15-26) | 0.69 |
| FIB-4 index | 4.35 (3.16-7.17) | 5.01 (2.94-7.48) | 0.51 |
| ALBI score | -2.51 (-2.82 — -2.19) | -2.44 (-2.73 — -2.15) | 0.43 |
| Fucosylated haptoglobin (×10^3^ relative unit) | 1.162 (0.952-1.387) | 2.308 (1.971-3.054) | <0.0001 |

Abbreviations: AFP, alpha-fetoprotein; ALBI, albumin-bilirubin; ALT, alanine aminotransferase; AST, aspartate transaminase; BMI, body mass index; DCP, des-γ- carboxy prothrombin; eGFR, estimated glomerular filtration rate; FBG, fasting blood glucose; FIB-4 index, fibrosis-4 index; GGT, γ- glutamyltransferase; HbA1c, hemoglobin A1c; HCV-RNA, hepatitis C virus-ribonucleic acid; PT, prothrombin time
