# Supplementary material for: Fucosylated haptoglobin is a novel predictive marker of hepatocellular carcinoma after hepatitis C virus elimination in patients with advanced liver fibrosis
Source: PLoS One. 2022 Dec 21;17(12):e0279416. doi: 10.1371/journal.pone.0279416 (PMC9770342; doi:10.1371/journal.pone.0279416)
Supplement: S3 Table — (DOCX) [file pone.0279416.s004.docx]

S3 Table. Characteristics of patients with high Fuc-Hp and low Fuc-Hp at the end of treatment

| Factor | Fuc-Hp ≤1330 relative unit (n=71) | Fuc-Hp >1330 relative unit (n=69) | p value |
| --- | --- | --- | --- |
| Sex: male/female (% male) | 37/34 (52.1%) | 24/45 (34.8%) | 0.043 |
| Platelets EOT (×10^4^/μl) | 13.2 (9.6-16.4) | 12.6 (9.3-15.3) | 0.53 |
| AST EOT (U/l) | 26 (21-37) | 32 (27-45) | 0.0005 |
| ALT EOT (U/l) | 18 (14-34) | 27 (20-37) | 0.0044 |
| GGT EOT (U/l) | 30 (20-39) | 31 (24-40) | 0.4 |
| Total bilirubin EOT (mg/dl) | 0.9 (0.7-1.1) | 0.7 (0.6-1.0) | 0.035 |
| eGFR EOT | 68.8 (58.3-79.0) | 71.7 (61.0-82.0) | 0.41 |
| FBG EOT (mg/dL) | 100 (92-114) | 101 (93-124) | 0.71 |
| HbA1c EOT (%) | 5.5 (5.1-5.8) | 5.6 (5.3-6.2) | 0.19 |
| Albumin EOT (g/dl) | 4.1 (3.8-4.4) | 4.0 (3.7-4.2) | 0.056 |
| PT EOT (%) | 85 (78-93) | 87 (78-93) | 0.76 |
| Hyaluronic acid EOT (ng/ml) | 148 (78-241) | 215 (119-344) | 0.0072 |
| Type 4 collagen 7S EOT (ng/ml) | 6.6 (5.5-7.6) | 7.3 (6.5-8.7) | 0.025 |
| AFP EOT (ng/ml) | 5 (3-9) | 6 (4-9) | 0.21 |
| DCP EOT (mAU/ml) | 18 (15-23) | 18 (15-21) | 0.88 |
| FIB-4 index EOT | 3.06 (1.96-4.50) | 3.61 (2.75-5.54) | 0.014 |
| ALBI score EOT | -2.76 (-2.97 — -2.48) | -2.65 (-2.86 — -2.43) | 0.13 |
| Fucosylated haptoglobin EOT (×10^3^ relative unit) | 0.911 (0.513-1.091) | 2.046 (1.66-2.8) | <0.0001 |

Abbreviations: AFP, alpha-fetoprotein; ALBI, albumin-bilirubin; ALT, alanine aminotransferase; AST, aspartate transaminase; DCP, des-γ- carboxy prothrombin; eGFR, estimated glomerular filtration rate; EOT, end of treatment; FBG, fasting blood glucose; FIB-4 index, fibrosis-4 index; GGT, γ- glutamyltransferase; HbA1c, hemoglobin A1c; PT, prothrombin time
